# Distribution of T Cells in Rainbow Trout (*Oncorhynchus mykiss*) Skin and Responsiveness to Viral Infection

**DOI:** 10.1371/journal.pone.0147477

**Published:** 2016-01-25

**Authors:** Esther Leal, Aitor G. Granja, Carlos Zarza, Carolina Tafalla

**Affiliations:** 1 Centro de Investigación en Sanidad Animal (CISA-INIA), Valdeolmos (Madrid), Spain; 2 Skretting Aquaculture Research Centre, PO Box 48, Stavanger, 4001, Norway; National Cheng Kung University, TAIWAN

## Abstract

Although the skin constitutes the first line of defense against waterborne pathogens, there is a great lack of information regarding the skin associated lymphoid tissue (SALT) and whether immune components of the skin are homogeneously distributed through the surface of the fish is still unknown. In the current work, we have analyzed the transcription of several immune genes throughout different rainbow trout (*Oncorhynchus mykiss*) skin areas. We found that immunoglobulin and chemokine gene transcription levels were higher in a skin area close to the gills. Furthermore, this skin area as well as other anterior sections also transcribed significantly higher levels of many different immune genes related to T cell immunity such as T cell receptor α (TCRα), TCRγ, CD3, CD4, CD8, perforin, GATA3, Tbet, FoxP3, interferon γ (IFNγ), CD40L and Eomes in comparison to posterior skin sections. In agreement with these results, immunohistochemical analysis revealed that anterior skin areas had a higher concentration of CD3^+^ T cells and flow cytometry analysis confirmed that the percentage of CD8^+^ T lymphocytes was also higher in anterior skin sections. These results demonstrate for the first time that T cells are not homogeneously distributed throughout the teleost skin. Additionally, we studied the transcriptional regulation of these and additional T cell markers in response to a bath infection with viral hemorrhagic septicemia virus (VHSV). We found that VHSV regulated the transcription of several of these T cell markers in both the skin and the spleen; with some differences between anterior and posterior skin sections. Altogether, our results point to skin T cells as major players of teleost skin immunity in response to waterborne viral infections.

## Introduction

The skin is the envelope that separates fish from the environment; however, it is not only a mechanical barrier, but a metabolically active organ, actually the largest organ in the fish. Thus, the skin plays a relevant role in protection, communication, sensory perception, locomotion, respiration, ion regulation excretion, and thermal regulation (reviewed by [1]). In general, teleost skin is composed of three layers: the mucous layer, the epidermis and the dermis. Because teleost skin is not keratinized, live skin cells are in close contact with the water medium and consequently can immediately react to pathogen exposure, being the skin (together with the gills) the first line of defense against waterborne pathogens. Despite this, there is a great lack of information regarding a phenotypic and functional characterization of the skin associated lymphoid tissue (SALT). Unlike mammals, fish lack major lymphoid accumulations in mucosal tissues; however the presence of different types of immune cells has been reported as part of the skin in many fish species [2]. Despite this, there is still a debate on whether these structures fulfill the definition of a lymphoid tissue and particularly of a true SALT.

Most fish express three different immunoglobulin (Igs) isotypes, IgM, IgT/Z and IgD and depending on the type(s) of Igs produced, different B lymphocyte subsets can be found. IgD^+^IgM^−^B cells have been reported in channel catfish (*Ictalurus punctatus*) [3] and in rainbow trout (*Oncorhynchus mykiss*) gills [4], but their role in immunity has not been yet clarified. On the other hand, IgD^+^IgM^+^ B cells are present in all teleost species analyzed thus far and represent the majority of B lymphocytes in fish. Finally, a lineage of B cells uniquely expressing IgT/Z has been reported in species such as rainbow trout [5] or zebrafish (*Danio rerio*) [6]. These cells seem to be mostly regulated at mucosal surfaces [5], although systemic responses have also been reported for them [7]. So far, secreted IgM and IgT have been described in mucosal skin secretions in fish [8, 9], but the presence of IgD has not been reported, even though a recent study has demonstrated the regulation of IgD in gills and intestine in response to immersion and oral vaccination, respectively [10]. Although IgM is the most common Ig in skin mucus, IgT seems to be the major Ig responder in rainbow trout skin upon infection with *Ichthyophtirius multifiliis* [9]. Interestingly, the response to this parasite is exclusively mediated by IgM in catfish, a species lacking IgT [8]. Then again, the presence of antigen secreting cells within the skin has also been demonstrated in catfish [11], while they remain to be fully characterized in other fish species such as rainbow trout.

T cells are characterized by the presence of a T cell receptor (TCR) by which they recognize antigens. Unlike B lymphocytes, T lymphocytes only recognize antigens when exposed in the context of an isogenic major histocompatibility complex (MHC), either class I or II. A first classification of T cells can be based on the TCR chains they express, either αβor γδ. αβ-T cells can be catalogued as conventional T cells whereas γδ-T cells recognize unprocessed antigens in a manner similar to that of pattern recognition receptors. Thus, in mammals, γδ-T cells are more innate-like immune cells, mostly present in epithelial and mucosal tissues, representing around 2% of the total T cell population [12]. On the other hand, conventional αβ-T cells can be divided into T cytotoxic (Tc) or T helper (Th) cells, distinguished by the expression of the membrane bound glycoproteins CD8 or CD4 respectively. These molecules act as co-receptors for the TCR, stabilizing the interaction with the MHC and enhancing TCR activation through CD3, present in all T lymphocyte subsets [5]. Tc cells are able to kill infected (mainly virus-infected) or cancerous cells after recognizing antigens in the context of MHC class I [13] through the release of effector molecules such as perforin or granzyme [14]. Th cells, on the other hand, express CD4 and produce cytokines to regulate the action of other immune cells, mainly B cells. In mammals, they are further classified according to the expression of specific transcription factors and the secretion of representative combinations of cytokines. Although there is still some controversy as to whether these Th subsets constitute differential cell lines or cells in a different stage of activation with a certain degree of plasticity [15], well-defined subsets in mammals include Th1, Th2, Th17 and Treg. The differentiation of Th cells towards a Th1 profile is controlled by the transcription factor Tbet [16]. These cells secrete effector cytokines such as interferon γ (IFNγ) and tumor necrosis factor α (TNF-α) to control intracellular infections, and interleukin 2 (IL-2) to induce lymphocyte proliferation. GATA3 is the transcription factor that mediates the differentiation of Th cells towards a Th2 profile [16]. Th2 cells produce IL-4, IL-5, and IL-13 that stimulate B cells and control extracellular infections through the secretion of antibodies. Th17 cells use the transcription factor RORγ and produce IL-17 together with IL-21 and IL-22 [17]. These cells appear to be implicated in the control of extracellular bacterial infections, although their precise role is still debated. Finally, Treg cells, which are regulated through Foxp3, have a crucial role in keeping self-tolerance [18]. Concerning fish, genomic studies performed in different species have identified most components associated with T cell function, making it possible to speculate that fish have all these different T cell subsets [19], however, whether the functionalities are maintained is something that needs to be further investigated. Although T cells have been identified in the intestinal mucosa [20], the presence of T lymphocytes in skin has not been investigated in depth in teleost fish. However, the transcription of several immune genes associated with T cell activities has been reported in skin suggesting that T lymphocytes are also components of the SALT in these species [21]. For instance, the transcription of GATA-3 and IL4/13, a homologue of the mammalian Th2 cytokine genes IL-4 and IL-13, was examined in several rainbow trout tissues, among which transcription levels were higher in thymus, skin and gills [22]. Similarly, the use of an anti- rainbow trout CD3 antibody, revealed high ratios of CD3^+^ cells in thymus, skin and posterior kidney, and lower ratios in head kidney, spleen, gills and peripheral blood leucocytes through flow cytometry techniques [23].

Finally, whether immune components of the skin are homogeneously distributed through the surface of the fish is still unknown, although some evidences indicate that the immune components of the skin are not equally distributed throughout the body surface. For example, in catfish, Ig levels were found to be highest immediately caudal to gill covers and between pectoral to anal fins, in comparison to the lower levels observed in anal to caudal skin and on ventral skin between the gill cover and pectoral fin [24].

In the current study, we have analyzed the levels of transcription of different immune genes throughout different skin sections. We have mainly focused in genes related to T lymphocyte responses, also studying the expression of Ig genes and some chemokines with a relevant mucosal role. Our results provide evidences that the skin is a T cell rich organ. Furthermore, as occurs with Ig expression, T cell immunity in the skin is also specially concentrated in the skin area immediately caudal to the gill cover and anterior skin areas. These results have been further confirmed by immunohistochemistry and flow cytometry. Finally, we have studied the transcriptomic response of T cells in the skin to a bath infection with viral hemorrhagic septicemia virus (VHSV), comparing this response to that observed in the spleen, the main secondary immune organ in fish. Although the skin is not a major target for VHSV replication, the virus is known to infect trout through the fin bases and to replicate in the skin [25], where it can regulate chemokine transcription [25] and induce the activation and mobilization of skin dendritic cells [26]. Similarly, in the current study, VHSV showed the capacity to regulate the transcription of genes related to T cell function in the skin. Our results reveal a major role of T lymphocytes in the response of the skin to waterborne viral pathogens.

## Materials and Methods

### Ethics statement

The experiments described comply with the Guidelines of the European Union Council (2010/63/EU) for the use of laboratory animals and were previously approved by the Ethics committee from the Instituto Nacional de Investigación y Tecnología Agraria y Alimentaria (INIA; Code CEEA 2011/044). Anesthesia was applied prior to sacrifice following the recommendations from Zhal *et al*. for general anesthesia (narcosis) [27]. All efforts were focused to minimize suffering.

### Fish

Healthy specimens of rainbow trout (*Oncorhynchus mykiss*) were obtained from the Centro de Acuicultura El Molino (Madrid, Spain). Fish were maintained at the Centro de Investigaciones en Sanidad Animal (CISA-INIA) laboratory at 16°C in 30 or 100 l rectangular tanks with a re-circulating water system and 12:12 hours light:dark photoperiod. Fish were fed twice a day with a commercial diet (Skretting, Spain). Prior to any experimental procedure, fish were acclimatized to laboratory conditions for 2 weeks and during this period no clinical signs were ever observed. The experiments described comply with the Guidelines of the European Union Council (86/609/EU) for the use of laboratory animals and were previously approved by the INIA Ethics committee.

### Tissue collection from naïve fish

**Fig 1:**
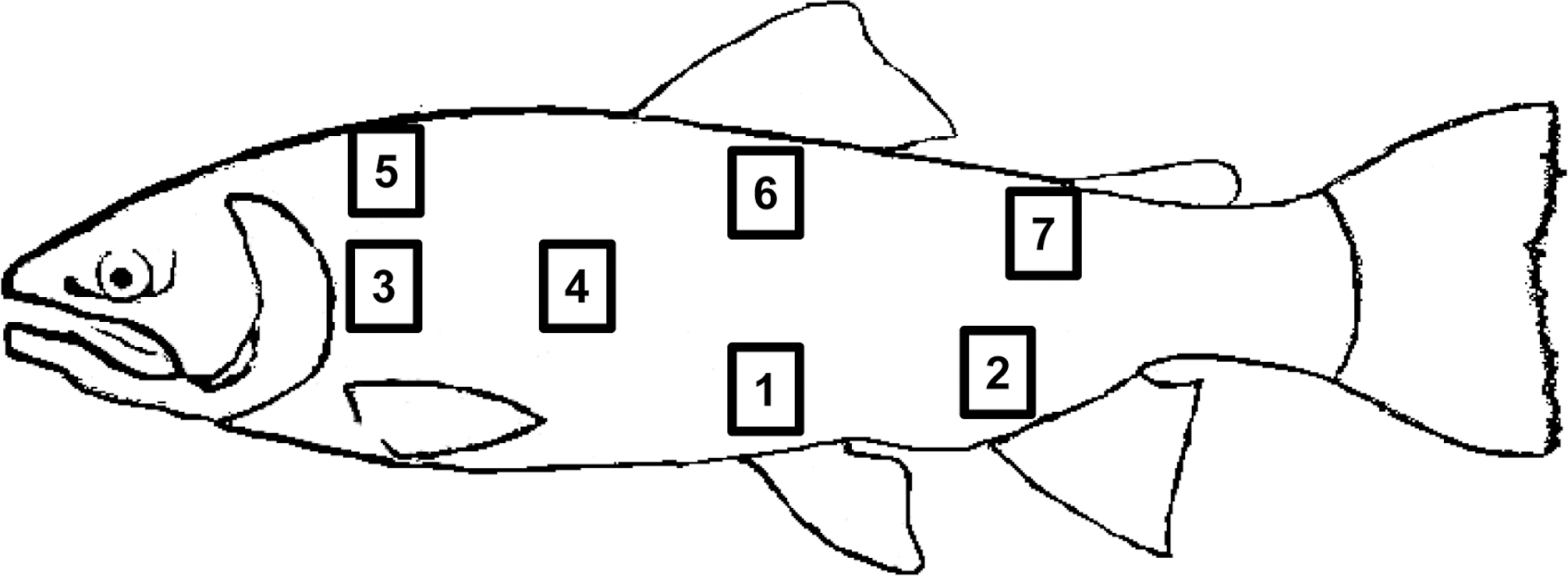
Skin sections sampled in this study. Illustration showing the distribution of the different skin sections used to study immune gene transcription in this study.

To characterize the transcriptional regulation of immune genes throughout different skin sections, unhandled naïve trout of approximately 8–10 cm were used. For this, seven different skin sections of 1 cm^2^ shown in Fig 1 were removed with a scalpel from fish previously sacrificed by benzocaine (Sigma) overdose (30 mg/l). The experiment was performed in 5 fish and was repeated once to make a final n of 10. The skin sections, all obtained from the same side, were further cleaned in sterile PBS 1x, removing all the muscle tissue present. Clean skin samples were then placed in Trizol reagent (Life Technologies) for RNA extraction. In some animals, for comparative purposes, the spleen was also removed and stored in Trizol for RNA extraction.

To study T cell distribution throughout fish skin by immunohistochemistry, naïve trout of approximately 3–5 cm were used. Fish were sacrificed by benzocaine overdose and then two transversal sections were obtained with a scalpel. One transversal section covered the area from the operculum to the end of the pectoral fins whereas the other section included the transversal section obtained from the beginning of the anal fin towards its end. The body fragments from three individual fish were immediately included in Bouin’s solution for further immunohistochemical analysis.

### RNA extraction and cDNA preparation

Total RNA was extracted from skin and spleen samples using a combination of Trizol reagent and RNAeasy Mini kit (Qiagen). In summary, samples were mechanically disrupted in 1 ml of Trizol using a disruption pestle. Then, 200 μl of chloroform were added and the suspension centrifuged at 12000 x *g* for 15 min. The clear upper phase was recovered, mixed with an equal volume of 100% ethanol and immediately transferred to RNeasy Mini kit columns. The procedure was then continued following manufacturer’s instructions, performing on-column DNase treatment. Finally, RNA pellets were eluted from the columns in RNase-free water and stored at -80°C until used. One μg of RNA was used to obtain cDNA in each sample using the Bioscript reverse transcriptase (Bioline Reagents Ltd) and oligo (dT)_12-18_ (0.5 μg/ ml) following manufacturer´s instructions. The resulting cDNA was diluted in a 1:4 proportion with water and stored at -20°C.

### Evaluation of immune gene expression by real time PCR

To evaluate the levels of transcription of the different genes, real-time PCR was performed in a LightCycler® 480 System instrument (Roche) using SYBR Green PCR core Reagents (Applied Biosystems) and specific primers previously optimized (shown in Table 1). Each sample was measured under the following conditions: 10 min at 95°C, followed by 45 amplification cycles (15 s at 95°C and 1 min at 60°C). The expression of individual genes was normalized to relative expression of trout EF-1α and the expression levels were calculated using the 2^-ΔCt^ method, where ΔCt is determined by subtracting the EF-1α value from the target Ct. Negative controls with no template were included in all the experiments. A melting curve for each PCR was determined by reading fluorescence every degree between 60°C and 95°C to ensure only a single product had been amplified.

**Table 1:** Primers used in this study.

Gene	Forward (5´-3´)	Reverse (5´-3´)
EF-1α	GATCCAGAAGGAGGTCACCA	TTACGTTCGACCTTCCATCC
CD3	CCTGATTGGAGTAGCTGTCTAC	GCTGTACTCAGATCTGTCCATGC
CD4	CCTGCTCATCCACAGCCTAT	CTTCTCCTGGCTGTCTGACC
CD8	AGTCGTGCAAAGTGGGAAAG	GGTTGCAATGGCATACAGTG
CD40L	GAGTGTGAGAAAGACAGCCAGTCAG	cgtttgacagcttttccttcaactt
CK9	AAGGCTCTTATGGGAACTGC	CCACTTCTGGCTGGGATTG
CK10	ATTGCCAAGATCCTCTTCTGTGTTC	CCTGAGGCTGGTAACCTATGACAAC
CK11	GAACATTCCTTTGAGCATACTAAT	TGCACAATACTTCCTCCCAT
CK12	GACATCGATGCCACTGTGTT	GGAGATGGTTCGCTCCAGAC
Eomes	ACAACGTATTTGTTGAGGTCGTGTT	CATCTTGTTACCTTGCATGTTGTTG
FoxP3	CCAGAACCGAGGTGGAGTGT	TGACGGACAGCGTTCTTCCA
GATA3	CCCATCGGTGCTAAACGAACA	GCTGTGGTGCTGCATTGCTT
IFNγ	GAAGGCTCTGTCCGAGTTCA	TGTGTGATTTGAGCCTCTGG
IgD	AGCTACATGGGAGTCAGTCAACT	CTTCGATCCTACCTCCAGTTCCT
IgT	AACATCACCTGGCACATCAA	TTCAGGTTGCCCTTTGATTC
IL4/13	CAACCCAACCAAAGATGAAGACG	CAACGGTGCACTTCTGAAGTTTG
IL17.1	CTGGCGGTACAGCATCGATA	GAGTTATATCCATAATCTTCGTATTCGGC
IL17.2	CTGGCGGTACAGCATCGATA	CAGAGTTATATGCATGATGTTGGGC
Perforin	GGAACGACGACCTGTTAGGA	TCATAGGGGAGGGCACATAG
RORγ	ACAGACCTTCAAAGCTCTTGGTT	GGGAAGCTTGGACACCATCTTTG
Secreted IgM	CCTTAACCAGCCGAAAGGG	TGAGGTTCTATCAATGGTTCTC
Tbet	GTTCTGCAGTCGCTTCATAAGTACC	CTATGAATTGGGTCTCTGGGAAGAC
TCRα	acgcacttggaattattcaacaaga	gcttcacatttctctgaaccaccta
TCRγ	gaggaagaacagacgaccagtatga	gacatggtggttggagtatcttttg
Total IgM	TGCGTGTTTGAGAACAAAGC	GACGGCTCGATGATCGTAAT

### Immunohistochemistry

Trout anterior and posterior body sections were fixed in Bouin’s solution for 24 h, embedded in paraffin (Paraplast Plus; Sherwood Medical) and sectioned at 5 μm. After dewaxing and rehydration, the sections were subjected to an indirect immunocytochemical method for detection of trout CD3° using a specific monoclonal antibody kindly donated by Erin Bromage from the University of Massachusetts Dartmouth (USA) [23]. After a heat induced epitope retrieval in Tris-EDTA buffer pH 9.0 (800 w for 5 min and 480 w for 5 min in a microwave oven), the sections were pre-incubated in two different blocking solutions consisting of 0.01% BSA (bovine serum albumin; Sigma) in TBT (Tris buffer containing 0.02% Tween-20 (Sigma)) at room temperature for 30 min, and 10% normal goat serum in TBT for 30 min. Sections were then incubated with primary antibody solution overnight at 4°C. Monoclonal mouse anti-trout CD3ε was used at a concentration of 10 μg/ml. Following this incubation, unbound primary antibodies were washed off using TBT. The tissue was covered with Polyclonal Goat Anti-Mouse Immunoglobulins/Biotinylated (Dako) used at a 1:300 dilution and then incubated for 15 min at room temperature. Subsequently, the tissue was washed three times with TBT and then incubated with Streptavidin HRP (Thermo Fisher Scientific) for 15 min. Afterwards the tissue was washed three times with TBT, and then incubated in AEC substrate [0.2M acetic acid buffer (pH 5) with sodium acetate 0.2 M, 0.015% H_2_O_2_ and 0.4 g/l 3-Amino-9-ethylcarbazole (Alfa Aesar)] for 8 min and later washed for 2 min in distilled water. The specificity of the reactions was determined by omitting the primary antibodies. Mayer’s haematoxylin (Dako) was used as nuclear counter stain, and mounting was conducted with Ultramount Aqueous Permanent Mounting Medium (Dako). Slides were examined with an Axiolab (Zeiss) light microscope.

### Flow cytometry

Skin sections taken from areas 5–3 (anterior) or 2–7 (posterior) as depicted in Fig 1 were used to obtain skin leukocytes following the protocol previously described [26]. Skin leukocytes obtained from these sections were incubated for 30 min with the primary antibody anti-trout CD8α (mAb rat IgG, 7 μg/ml), washed twice with staining buffer (PBS containing 1% FCS and 0.5% sodium azide) and stained for 20 min with R-phycoerythrin F(ab')_2_ fragment of goat anti-rat IgG (H+L) (Life Technologies). After incubation, cells were washed three times with staining buffer, and analyzed on a FACSCalibur flow cytometer (BD Biosciences) equipped with CellQuest Pro software. Flow cytometry analysis was performed with FlowJo 10 (TreeStar).

### VHSV *in vivo* infection

Fish of about 5–7 cm were transferred to 4 l of a viral solution containing 5 x 10^5^ TCID_50_/ml of the VHSV strain 0771. Mock-infected groups were also transferred to 4 l tanks containing an equivalent amount of non-infected culture media. After 1 h of viral adsorption with strong aeration at 16°C, each experimental group was transferred to an individual 30 l water tank. At days 1 and 3 post-infection, six trout from each group were sacrificed by over-exposure to benzocaine and skin (sections 3 and 7 as indicated in Fig 1) and spleen were sampled and placed in Trizol for RNA isolation following the method described above.

### Statistics

Data handling, analyses and graphic representation was performed using Microsoft Office Excel 2010. Statistically significant differences were determined using an ANOVA test (p<0.05) using the Sigma Stat (Version 3.5).

## Results

### Transcription of chemokine and Ig genes throughout the trout skin

**Fig 2:**
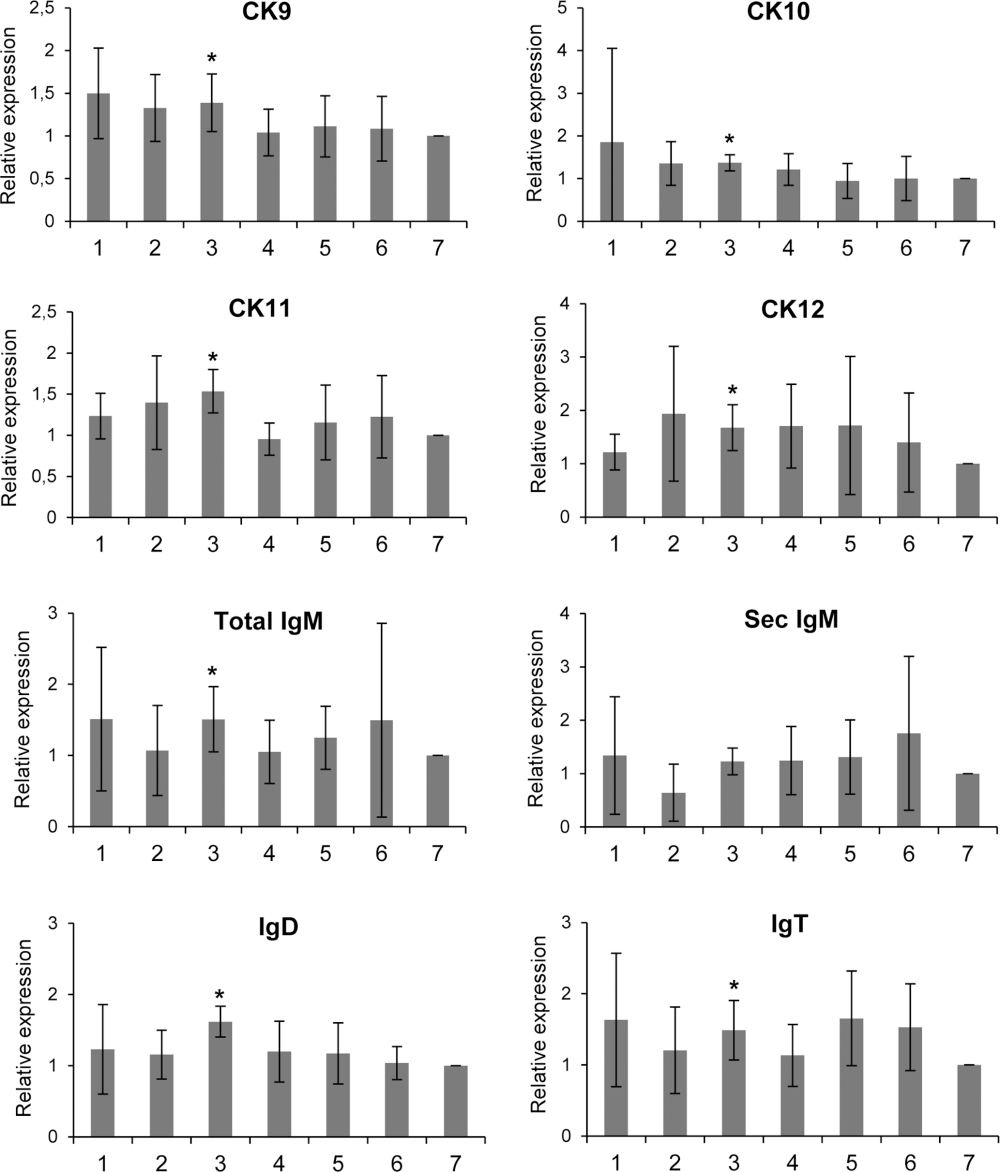
Constitutive levels of expression of chemokines and genes related to B cell activity in the different skin sections. The skin sections indicated in Fig 1 were sampled from naïve fish for RNA extraction and analysis of immune gene transcription through real time PCR. Data are shown as the mean relative gene expression normalized to the transcription of the house-keeping gene EF-1α ± SD (n = 10). Relative expression values were lowest in section 7 for most genes analyzed and were set as 1 for comparative reasons. Asterisks indicate significant differences (*P* < 0.05) relative to values in section 7.

We first studied whether different chemokine genes were homogenously distributed throughout the different skin sections depicted in Fig 1. For this study, we chose CK9, CK10, CK11 and CK12 chemokines as it has been previously shown that they are strongly expressed in mucosal tissues [25]. All four chemokines studied were expressed at significantly higher levels in section 3, a skin area close to the gills, when compared to section 7, which was the more posterior region sampled used for normalization given the fact that the majority of the immune genes analyzed in this study had lower expression levels in this section (Fig 2). Similarly, total IgM, IgD and IgT transcription was also higher in section 3 when compared to the posterior section 7 (Fig 2). On the other hand, the transcription levels of secreted IgM were not significantly different throughout the skin sections sampled due to a high individual variation (Fig 2).

### Transcription of genes related to T cell immunity throughout the trout skin

**Fig 3:**
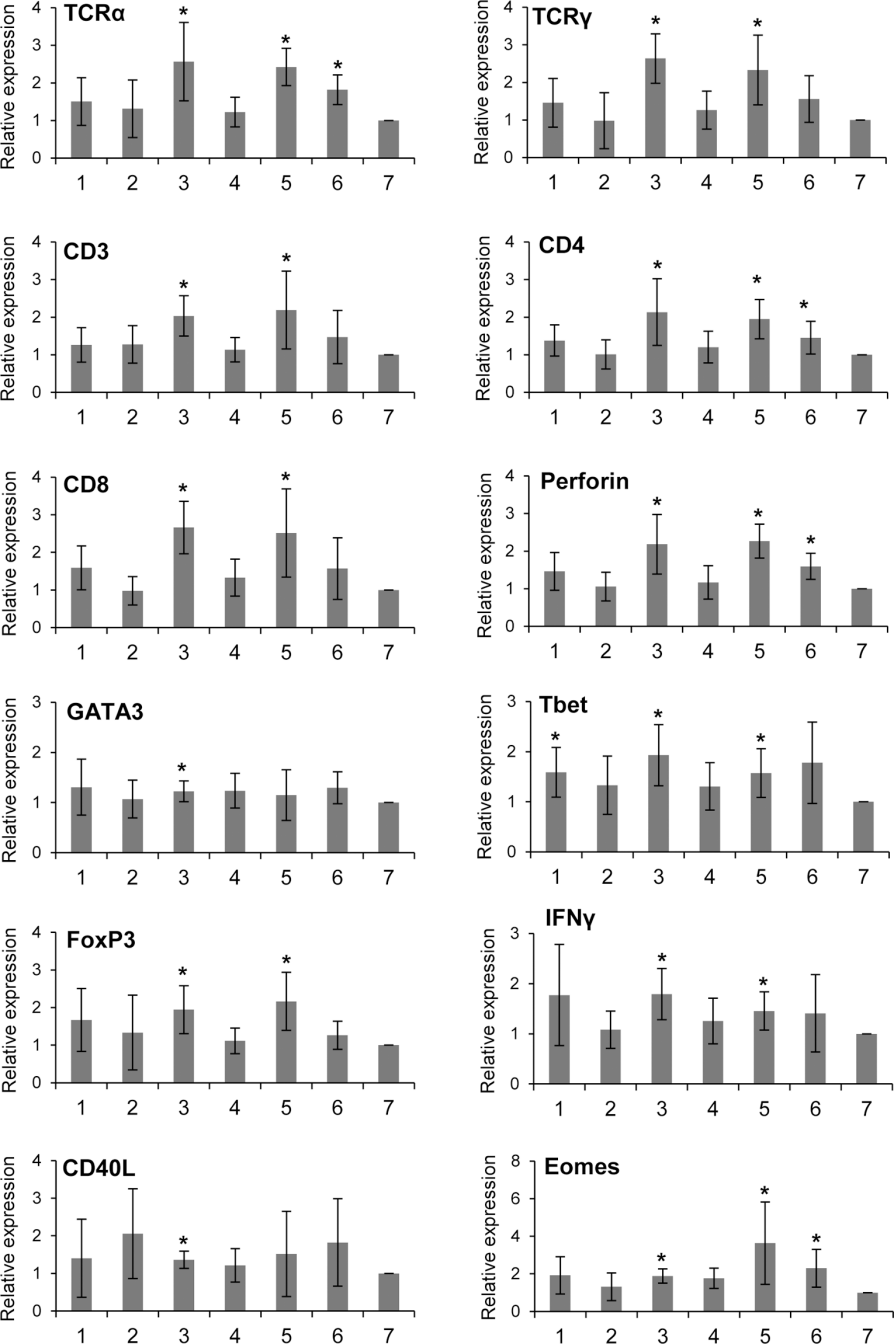
Constitutive levels of expression of genes related to T cell activity in the different skin sections. The skin sections indicated in Fig 1 were sampled from naïve fish for RNA extraction and analysis of immune gene transcription through real time PCR. Data are shown as the mean relative gene expression normalized to the transcription of the house-keeping gene EF-1α ± SD (n = 10). Relative expression values were lowest in section 7 for most genes analyzed and were set as 1 for comparative reasons. Asterisks indicate significant differences (*P* < 0.05) relative to values in section 7.

When we studied the transcription of several immune genes related to T cell immunity throughout the different skin sections, we observed that, again, the levels of transcription were significantly higher in section 3 when compared to section 7 (Fig 3, S1 Fig). This was true for all genes studied, namely, T cell receptor α (TCRα), TCRγ, CD3, CD4, CD8, perforin, GATA3, Tbet, FoxP3, interferon γ (IFNγ), CD40L and Eomes, the latter being a key molecule associated with the function and differentiation of CD8^+^ T cells [28]. In the case of TCRα, TCRγ, CD3, CD4, CD8, perforin, Tbet, FoxP3 and IFNγ, transcription levels were also higher in the anterior section 5 in comparison to the reference section 7 (Fig 3). This uneven distribution of T lymphocyte marker genes was visualized in all individual fish analyzed (S1 Fig) and suggests that all anterior sections, not only those close to the gill operculum, are rich in T cells. Likewise, a strong correlation of transcription levels between different T cell marker genes in the different skin sections (1, 5 and 7) further supports this statement (S2 Fig). Surprisingly, mRNA levels for the different T cell related genes in section 4 were not as high as expected from its anterior-posterior position, which might indicate a lower number of immune cells along the lateral line. Finally, some of these genes were also maintained at significantly higher levels in sections 1 (Tbet) or 6 (TCRα, CD4 and perforin) in comparison to section 7 (Fig 3).

### Distribution of T cells throughout the skin

**Fig 4:**
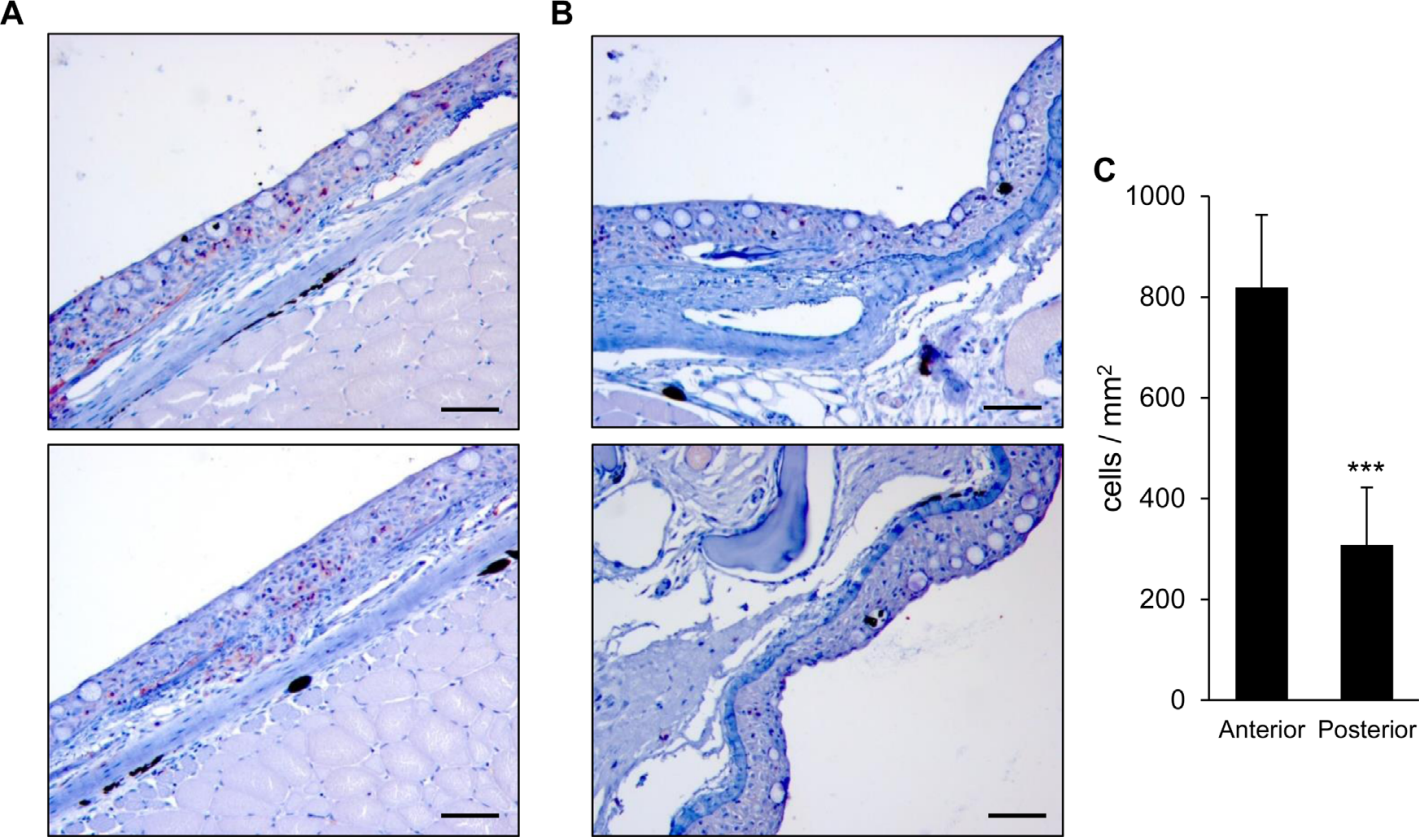
Distribution of trout CD3^+^ cells in anterior and posterior skin regions. Inmunohistochemical detection of trout CD3^+^ cells in different skin areas of anterior (A) and posterior (B) body sections. Counterstained with Mayer’s haematoxylin. Scale bar represents 100μm. Total numbers of CD3^+^ cells for each skin section were calculated using the imaging analysis Image J software (NIH). Then, relative number of cells per mm^2^ were calculated and plotted. Data are shown as mean ± SD (n = 10). Asterisks indicate mean values in posterior sections significantly lower than values in anterior areas (*P* < 0.001).

Our transcriptional studies suggested that the anterior skin sections were richer in T cells, and to confirm this hypothesis, we conducted an immunohistochemical analysis of CD3^+^ cells in histological samples obtained from anterior and posterior transversal body sections. We found indeed that the number of CD3^+^ T cells within the skin was higher in anterior body sections (Fig 4A and 4C) in comparison to the corresponding posterior body sections (Fig 4B and 4C). In anterior skin areas, multiple CD3^+^ T cells were observed within the epidermis, whereas only scattered T cells could be detected in posterior skin sections. In some anterior skin areas CD3^+^ cells were also identified along the basal layer (Fig 4A).

**Fig 5:**
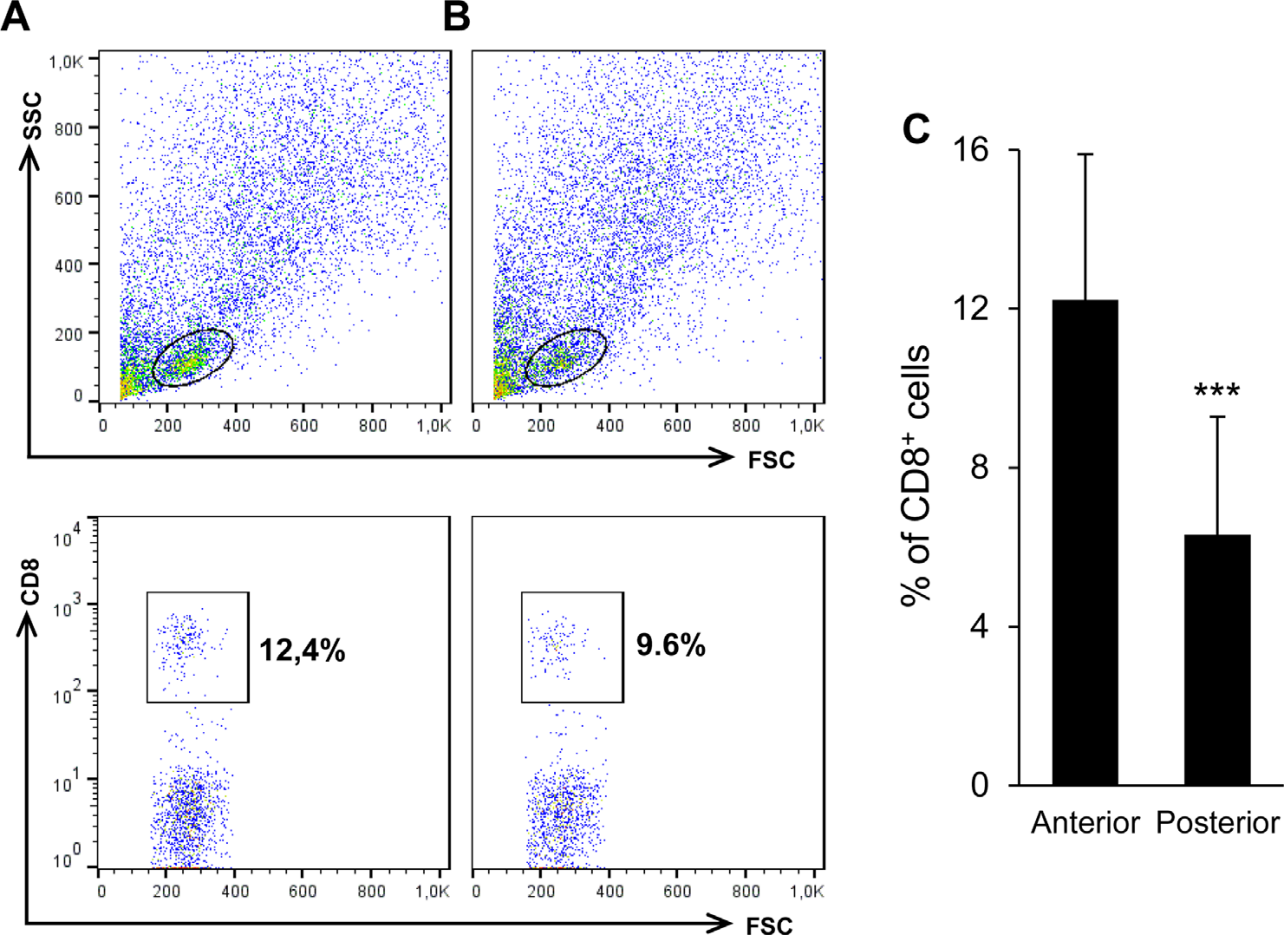
Quantification of trout CD8^+^ cells in anterior and posterior skin regions. Flow cytometry analysis of rainbow trout leukocytes isolated from anterior (A) and posterior (B) sections of the skin were stained with anti-CD8α mAb. For each individual tissue, FSC/SSC profiles are shown (top panels) and gates for lymphoid cells were defined. Bottom panels depict the CD8^+^ cells among the lymphoid gate (percentages of CD8α^+^ cells are shown). (C) Mean percentages of CD8α^+^ cells from three independent experiments are shown (n = 9 fish, mean ± SD). Asterisks indicate mean values in posterior sections significantly lower than values in anterior areas (*P* < 0.001).

Additionally, we studied the relative percentage of CD8^+^ T lymphocytes in anterior and posterior skin sections through flow cytometry, confirming that the percentage of this specific T lymphocyte subset is significantly higher in anterior sections, reaching 12% of the lymphocyte population, while the percentage of CD8^+^ cytotoxic T cells was around 6% in the most posterior skin sections (Fig 5). On the other hand, these percentages of CD8^+^ T lymphocytes visualized in the skin are much higher than those previously reported for spleen (around 2.3% of the lymphocyte population) [29].

### Comparison of T cell related transcription levels in skin and spleen

**Fig 6:**
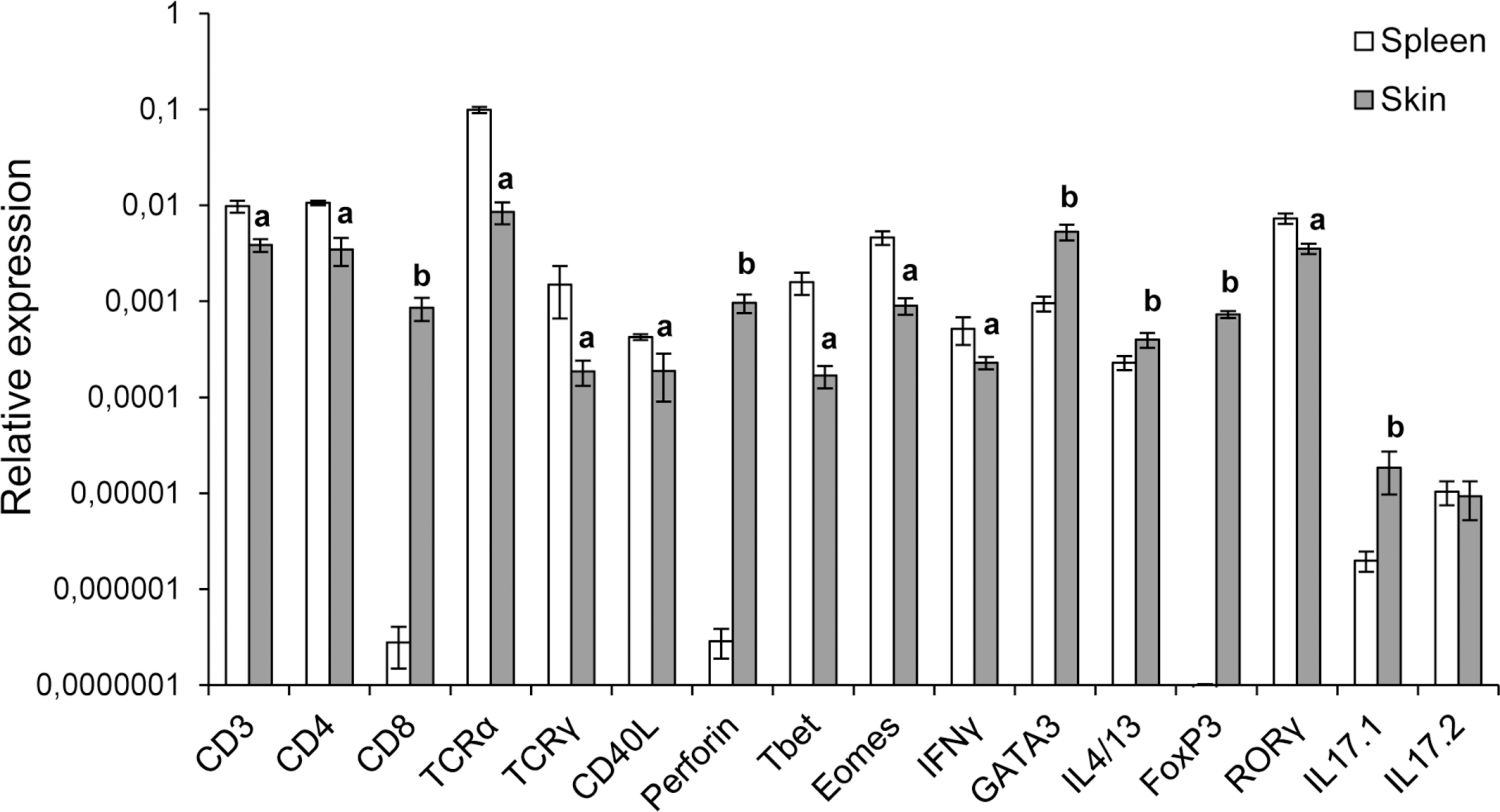
Comparative transcription of genes related to T cell activity in skin and spleen. The levels of transcription of different genes related to T cell activity were studied in skin samples corresponding to section 3 as indicated in Fig 1 and spleen in unstimulated fish through real time PCR. Data are shown as the mean relative gene expression normalized to the transcription of the house-keeping gene EF-1α ± SD (n = 5). “a” indicate values in skin significantly lower to values obtained in spleen whereas “b” indicate skin values significantly higher than those found in spleen (*P* < 0.05).

To confirm that the skin, and specially the anterior section, is a tissue rich in T cells, we compared the levels of transcription of the different genes related to T cell immunity in the anterior skin section to those observed in the spleen of naïve fish. Although the levels of transcription of CD3, CD4, TCRα, TCRγ, CD40L, Tbet, Eomes, IFNγ and RORγ were significantly higher in spleen than in the skin, the high levels of transcription observed for all these genes suggested an important T cell presence (Fig 6). Interestingly, the higher CD8 and perforin transcription levels detected in the skin appear to indicate that the presence of cytotoxic T cells is higher in skin than in the spleen (Fig 6), in concordance with our flow cytometry results. This also seems to be the case for Treg cells and Th17 cells, since FoxP3 and IL17.1 levels are also higher in the skin than in spleen (Fig 6). Finally, the higher mRNA levels of GATA3 and IL4/13 observed in the skin also point to a subset of T cells skewed towards a Th2 profile in this tissue, as was previously suggested [22].

### Effect of VHSV on the transcription of T cell related genes in skin

**Fig 7:**
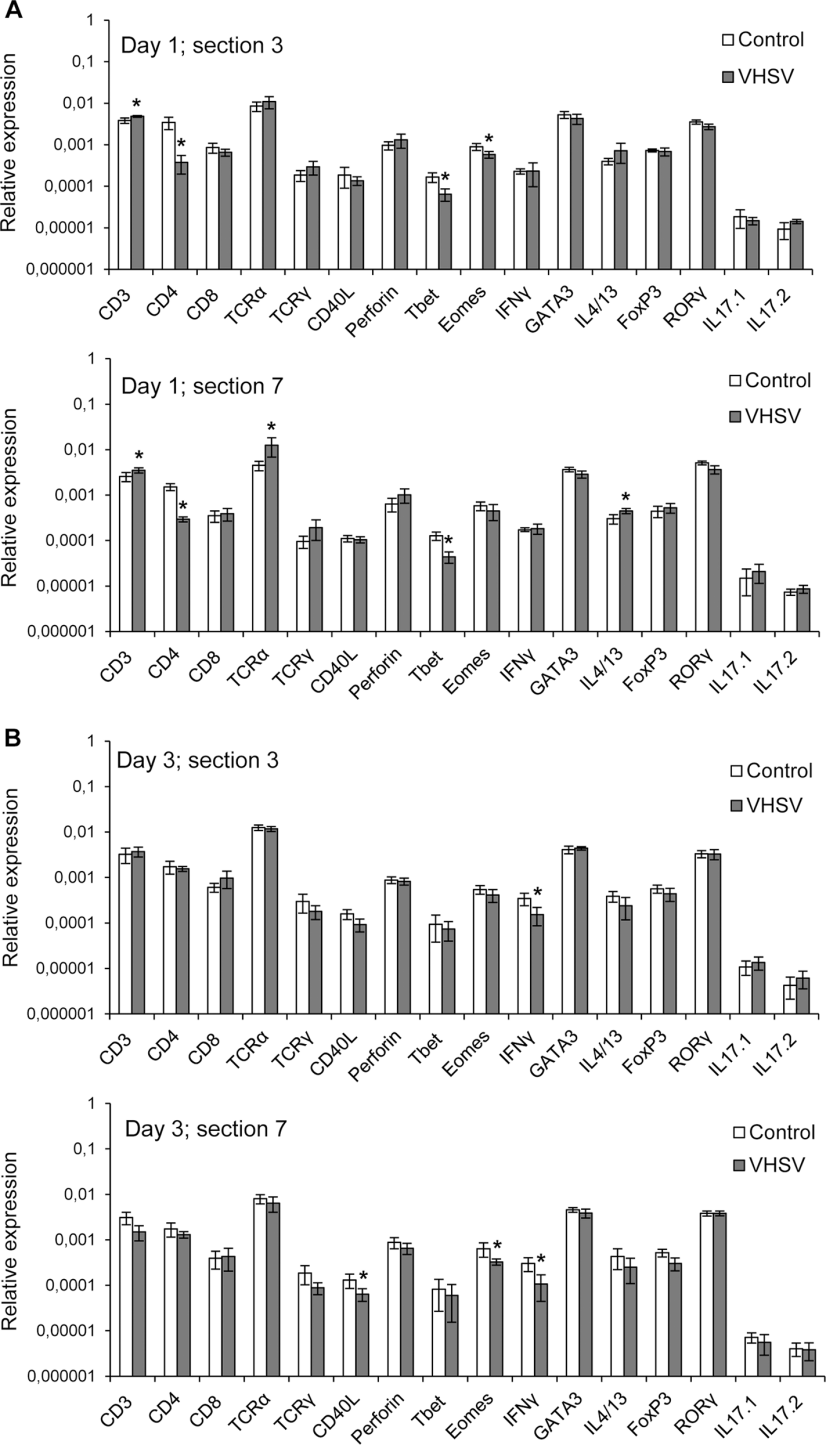
Transcription levels of immune genes characteristic of T lymphocytes in skin in response to VHSV. Rainbow trout were infected by bath with VHSV (5 x 10^5^ TCID_50_/ml) or mock-infected. At days 1 (A) and 3 (B) post-infection, six trout from each group were killed and the two sections in the skin corresponding to those indicated as 3 and 7 in Fig 1 sampled to determine the levels of expression of a selection of immune genes related to T cell immunity by real-time PCR. Data are shown as the mean gene expression relative to the expression of endogenous control EF-1α ± SD (n = 5). * Levels of expression in VHSV-infected animals significantly different to those observed in mock-infected fish (p < 0.05).

Because T cells are key players in the immune response against viral infections, we next studied how a bath infection with VHSV affected the levels of transcription of these genes related to T cell immunity in the skin. For this study we chose to analyze the responses in sections 3 and 7 as representatives of an anterior and a posterior skin area respectively. At day 1 post-infection, we observed a significant up-regulation of CD3 both in anterior and posterior sections suggesting T cell activation in response to the virus (Fig 7A). In section 7, an increase in TCRα was also detected at day 1 post-infection (Fig 7A). On the other hand, VHSV provoked a significant decrease of CD4 and Tbet in both areas that along with an increase in IL4 and the decrease in IFNγ mRNA levels at day 3, might imply a decline in the number of Th1 cells in the skin in response to the virus (Fig 7B). The decrease in Eomes transcription levels observed in anterior skin sections at day 1 and in posterior skin sections at day 3 also suggest a negative effect of VHSV on CD8^+^ T cytotoxic populations (Fig 7B). Our results suggest that in fish early T cell responses to waterborne viral infections are initially triggered at a local level in the mucosa.

### Effect of VHSV on the transcription of T cell related genes in spleen

**Fig 8:**
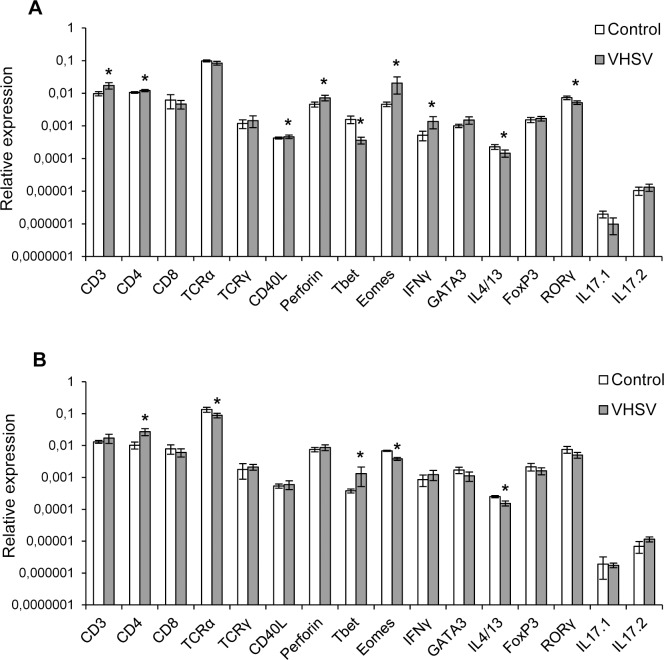
Transcription levels of immune genes characteristic of T lymphocytes in spleen in response to VHSV. Rainbow trout were infected by bath with VHSV (5 x 10^5^ TCID_50_/ml) or mock-infected. At days 1 and 3 post-infection, six trout from each group were killed and the spleen sampled to determine the levels of expression of a selection of immune genes related to T cell immunity by real-time PCR. Data are shown as the mean gene expression relative to the expression of endogenous control EF-1α ± SD (n = 5). * Levels of expression in VHSV-infected animals significantly to those observed in mock-infected fish (p < 0.05).

To understand whether the T cell responses mounted in the spleen to waterborne viral infections were comparable to those of the skin, we studied the effect of VHSV on the levels of expression of immune genes related to T cell immunity in the spleen. In this case, a significant increase in CD3 and CD4 transcription levels was observed in response to the virus at day 1 post-infection (Fig 8A) and also at day 3 in the case of CD4 (Fig 8B). Although Tbet transcription decreased in response to the virus at day 1 post-infection, it significantly increased in the infected spleen at day 3 post-infection. This effect, together with the significant increase in IFNγ mRNA levels observed at day 1, suggests an activation of Th1 responses in the spleen in response to the virus that also correlates with the significantly decreased IL4/13 levels observed at both days 1 and 3 post-infection (Fig 8A, B). The increased Eomes and perforin levels detected in viral infected spleens at day 1 also suggest an activation of T cytotoxic cells. Finally, the significant decrease in RORγ transcription observed at day 1 reveals a negative regulation of Th17 populations.

## Discussion

In the current study, we have established that teleost skin is a T rich tissue, with transcription levels of T cell related factors and T cell presence comparable to those found in the spleen. This seems to indicate that teleost fish mucosa, in the absence of lymph nodes, behave as secondary lymphoid organs where B and T cells coincide and are directly activated. These CD3^+^ T cells are located directly in the epidermis of rainbow trout, as previously reported for IgM^+^ B cells [30]. The presence of CD8^+^ cells in rainbow trout epidermis had already been informed, however, it should be noted that we have recently identified a population of dendritic cells in rainbow trout skin that expresses CD8 on the cell surface [26], thus when CD8 is used as a T cell marker, only cells with lymphocyte-like morphology have to be taken into account. On the other hand, CD3 is a T cell specific pan-T lymphocyte marker.

A previous study performed in rainbow trout demonstrated that GATA3 and IL4/13 transcription levels were higher in skin, gills and thymus when compared to spleen [22]. These results were used to hypothesize that the T environment in the skin is skewed towards a Th2 profile. Our results confirm this hypothesis as we also observed higher GATA3 and IL4/13 transcription levels in skin compared to levels found in the spleen. Additionally, we found that CD8 and perforin mRNA levels were also much higher in skin than in spleen, revealing an important presence of CD8^+^ T cells in this tissue. In correlation with these results, the percentage of CD8^+^ cytotoxic T lymphocytes in the skin as determined by flow cytometry was much higher that the percentages previously reported for this T lymphocyte subset in the spleen [29]. Similarly, FoxP3 and IL17.1 transcription values were also much higher in the skin than in the spleen, also suggesting an important presence of Treg and Th17 cells in the periphery, probably to maintain peripheral tolerance. Therefore, it seems that most T lymphocyte subtypes are located in teleost epidermis in physiological conditions, waiting to be activated in response to waterborne pathogens.

Interestingly, we have demonstrated that the location of these T cells throughout the epidermis is not homogenously distributed throughout the fish surface, but that the most anterior skin sections are the ones mostly enriched in T cells. This was confirmed by immunohistochemistry, flow cytometry and real-time PCR, observing that all T cell signature genes examined showed this precise pattern of expression which suggests that the anterior skin sections are enriched in all types of T cell subsets and not just in one in particular. The reason for this distribution of immune cells throughout the body surface is unknown but could be the consequence of the anterior location of the thymus and the proximity of a gill interbranchial-associated lymphoid tissue (GIALT) where T cells are known to assemble in teleost fish besides the thymus and the spleen [31]. Thus, because T cells should colonize the skin from these primary lymphoid tissues, this specific distribution of cells in the skin is produced. It could be then possible that B lymphocytes are attracted to T-rich areas, so antigen presentation and an initial trigger of the local immune responses are facilitated. Accordingly, we found that the transcription of IgM, IgT and IgD was also higher in the anterior section close to the operculum where T cell enrichment is greater, in concordance to previous results that demonstrated that IgM in the mucus was highest next to gill covers [24]. This migration of B lymphocytes to T rich areas could be mediated through the action of specific chemokines, since we also observed increased chemokine transcription in this area. Although this hypothesis needs to be confirmed, we do know that some of these highly expressed chemokines have the capacity to attract B lymphocytes [32]. These immune distribution in the skin should definitely have consequences in the way fish respond to waterborne pathogens and in this sense it is interesting to note that some diseases affecting teleost skin show a particular pattern that rarely involves the most anterior body sections [33]. For example, puffy skin disease (PSD) is a disease of unknown ethology that affects rainbow trout, producing cutaneous swelling, pigment loss and petechiae. PSD lesions have been reported to start in the middle third of the flank, never in the most anterior third and always involving the lateral line [34, 35], where we also observed less T cell related factor transcription.

Having established that T cells are a major component of the teleost SALT, we then studied how an infection with a virus could modulate the levels of transcription of these T cell related factors in the skin. We observed that the virus modulated many of these T related factors, demonstrating a responsiveness of skin T cells to viral infections, although differences were observed depending on the origin of the skin sample analyzed and the time at which it was sampled. At day 1 post-infection, although an increase in CD3 levels was observed suggesting some migration of T cells towards the skin, CD4 transcription levels were drastically reduced. This decrease in CD4 mRNA levels should correlate with a decrease in the number of CD4^+^ T cells as CD4 is not transcriptionally regulated in Th cells [36]. The decrease in the levels of transcription of Tbet, CD40L and IFNγ also produced in response to VHSV seem to support this hypothesis. Interestingly, CD4, Tbet and IFNγ are up-regulated in the spleen during this early time points, suggesting a mobilization of Th1 cells from the skin to the spleen. The skew of spleen T cell responses towards a Th1 response will surely be beneficial for the host since Th1 cells have been shown to play a crucial role in the elimination of viral pathogens inducing the production of virus-neutralizing specific antibodies by specific B cells and enhancing the activity of cytotoxic T cells [37]. Notably, the function of cytotoxic T cells also seems regulated in response to VHSV since Eomes transcription also is down-regulated in the skin and up-regulated in the spleen at day 1 post-infection, along with perforin.

The regulation of T cell factors in the skin in response to waterborne infections should be specific depending on the pathogen encountered; however, diverse parasitic infections also seem to modulate T cell responses in the skin, in addition to viruses. For example, IFNγ levels in the skin increased in rainbow trout upon infection with *Gyrodactylus salaris* although in this case other T related genes such as CD4 or CD8 were not significantly modulated by the infection [38]. On the other hand, *Ichthyobodo necator* also provoked an increase of IFNγ, but in this case, GATA3 and CD4 mRNA levels were also significantly up-regulated [30]. All these studies, along with our results, highlight the importance of T cell activity in the skin.

In conclusion, we have provided strong evidence to demonstrate that the teleost epidermis is a T lymphocyte rich tissue and that T cell responses are regulated in response to viral infections at very early time points in the skin as well as in the spleen. We have also demonstrated for the first time in teleost that T cells are not homogeneously distributed throughout the skin, but that anterior skin sections, especially those close to the operculum, are particularly enriched in T cells. Our results will be important for future studies aimed at understanding how pathogens and immersion vaccines modulate the immune response of the skin in teleost.

## Supporting Information

S1 Fig Constitutive levels of expression of T cell related genes in the anterior sections 1 and 5, and the posterior section 7. Results obtained in Fig 2 for CD3, CD4, CD8, TCRα, TCRγ and perforin were plotted as individual dispersion charts for each gene, for sections 1, 5 and 7, in order to visualize differences of expression of the analyzed genes between sections in the same individual. Data are shown as the mean relative gene expression normalized to the transcription of the house-keeping gene EF-1α (n = 10). Each fish is represented by a different symbol.
(PPTX)

S2 Fig Correlation between the expressions of T cell related genes in the anterior sections 1 and 5, and the posterior section 7. Results obtained in Fig 2 for CD3, CD4, CD8, TCRα, TCRγ and perforin were plotted as correlative dispersion chart, for sections 1, 5 and 7. Correlated parameters were: CD3 vs TCRα, CD3 vs TCRγ, CD3 vs CD4, CD3 vs CD8, CD3 vs Perforin and CD8 vs Perforin. For each XY dispersion chart, a linear regression trend line is shown, together with the value of the correlation coefficient, denoted by R. Data are shown as the mean relative gene expression normalized to the transcription of the house-keeping gene EF-1α (n = 10).
(PPTX)
