# Supplementary material for: Distribution of T Cells in Rainbow Trout (Oncorhynchus mykiss) Skin and Responsiveness to Viral Infection
Source: PLoS One. 2016 Jan 25;11(1):e0147477. doi: 10.1371/journal.pone.0147477 (PMC4726708; doi:10.1371/journal.pone.0147477)
Supplement: S1 Fig — Results obtained in Fig 2 for CD3, CD4, CD8, TCRα, TCRγ and perforin were plotted as individual dispersion charts for each gene, for sections 1, 5 and 7, in order to visualize differences of expression of the analyzed genes between sections in the same individual. Data are shown as the mean relative gene expression normalized to the transcription of the house-keeping gene EF-1α (n = 10). Each fish is represented by a different symbol. (PPTX) [file pone.0147477.s001.pptx]

## Slide 1
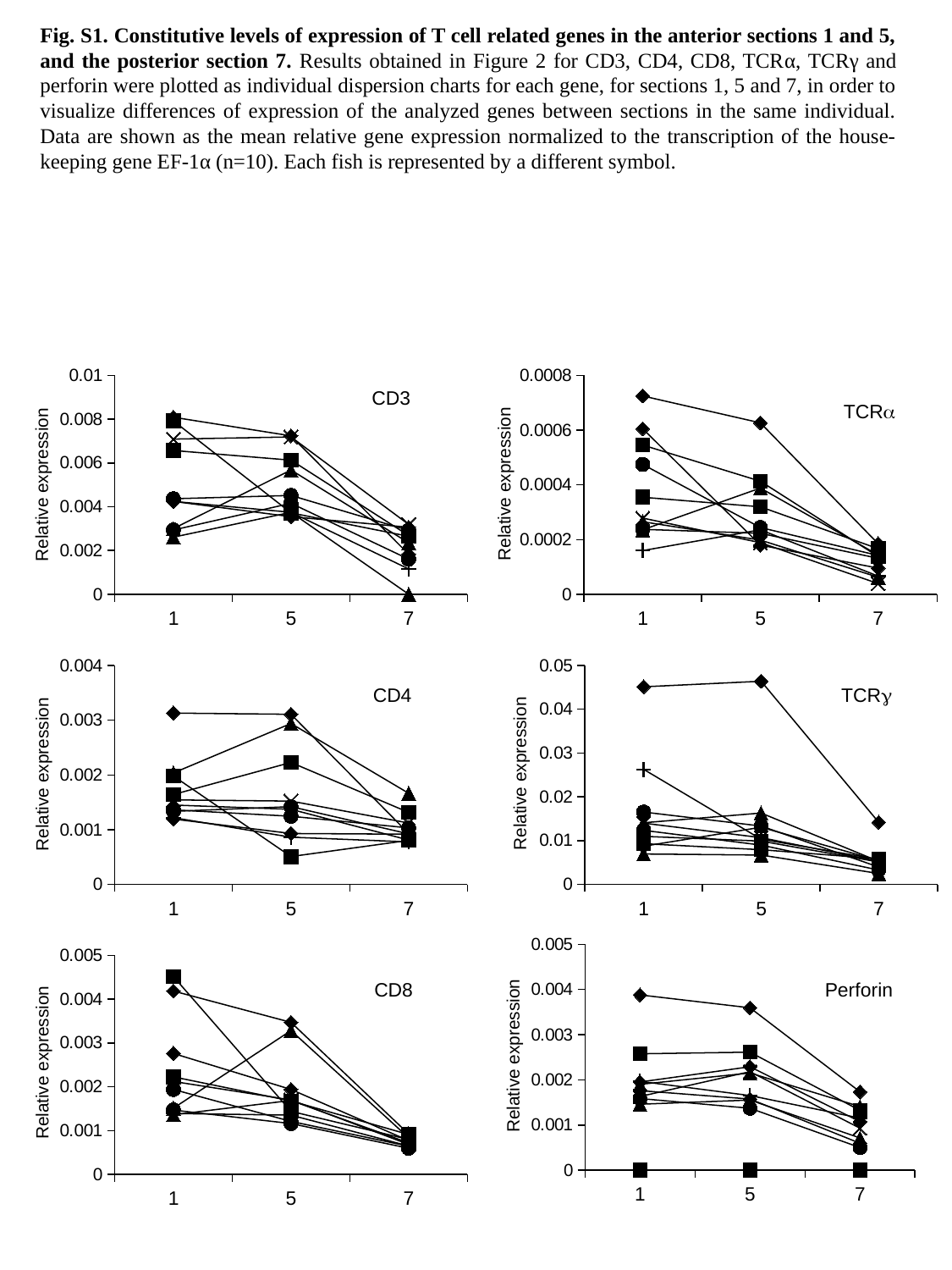

Fig. S1. Constitutive levels of expression of T cell related genes in the anterior sections 1 and 5, and the posterior section 7. Results obtained in Figure 2 for CD3, CD4, CD8, TCRα, TCRγ and perforin were plotted as individual dispersion charts for each gene, for sections 1, 5 and 7, in order to visualize differences of expression of the analyzed genes between sections in the same individual. Data are shown as the mean relative gene expression normalized to the transcription of the house-keeping gene EF-1α (n=10). Each fish is represented by a different symbol.
### Chart
| Category | | | | | | | | | | |
|---|---|---|---|---|---|---|---|---|---|---|
| 1 | 0.008088007217510768 | 0.003001709338453151 | 0.0026131397554416246 | 0.0042450580567424175 | 0.0029399350535372452 | 0.0043644028830946095 | 0.006569503244169644 | 0.007089993400915326 | 0.007921558435859595 | 0.0042450580567424175 |
| 5 | 0.007238969233518536 | 0.005679580145782461 | 0.0037471254661143125 | 0.0037471254661143186 | 0.004157696025208433 | 0.004518313218380027 | 0.006129563264818369 | 0.007188966020506835 | 0.003721242179859134 | 0.0035449967004576627 |
| 7 | 0.0018350053695586157 | 0.0023550934134585126 | 8.465343262236365e-06 | 0.0011613350732448465 | 0.0016085762056007283 | 0.0029196274387401156 | 0.0026866051135541868 | 0.003194929916241334 | 0.0026496178270462327 | 0.0030647816324091844 |
### Chart
| Category | | | | | | | | | | |
|---|---|---|---|---|---|---|---|---|---|---|
| 1 | 0.0007248650247212154 | 0.0001599601323069293 | 0.00047492917354115494 | 0.00027850676656943974 | 0.00023582429905391746 | 0.00035497375911140396 | 0.0005455503603868265 | 0.00023746458677057787 | 0.000265316128546401 | 0.0006053260253608334 |
| 5 | 0.0006266728015443873 | 0.00023582429905391746 | 0.0002441406250000002 | 0.0001889117423757798 | 0.0003884455487084564 | 0.0003199202646138581 | 0.0004134498595520151 | 0.00022310338140366223 | 0.00019830380770415902 | 0.00017996450406385537 |
| 7 | 0.00018502399493535145 | 6.679038703984015e-05 | 0.00014217939287945034 | 3.9439478474044585e-05 | 0.00014416414324090946 | 0.00016675296102958948 | 0.00013829148990427255 | 0.00013174172808891945 | 6.275108805273842e-05 | 9.577442601278705e-05 |CD3
TCRa
Relative expression
Relative expression
### Chart
| Category | | | | | | | | | | 1 |
|---|---|---|---|---|---|---|---|---|---|---|
| 1 | 0.00312917920933446 | 0.0020360659391428187 | 0.001449730049442431 | 0.0016423758110424135 | 0.0013620543616703184 | 0.0013340236882367136 | 0.001219072801661731 | 0.001543049437233157 | 0.0011939846461836614 | 0.0019803896089649036 |
| 5 | 0.0031075643896676516 | 0.0029399350535372452 | 0.0013715281989628864 | 0.0022280541325555145 | 0.0012446881126164664 | 0.0014198950364456174 | 0.0008620146448150939 | 0.0015218058196494143 | 0.0009303105449647817 | 0.0005090164847857037 |
| 7 | 0.0009303105449647817 | 0.0016653025229842909 | 0.000809882368959417 | 0.0013156577899079006 | 0.0010322441802357234 | 0.0009238844206304653 | 0.0007768910974169128 | 0.001121775737301792 | 0.0009175026847793079 | 0.0008042881028003648 |
### Chart
| Category | | | | | | | | | | | |
|---|---|---|---|---|---|---|---|---|---|---|---|
| 1 | 0.04512278736007804 | 0.006944083446613831 | 0.013984766733249552 | 0.009290680585958758 | 0.012344395497865271 | 0.008728805766189235 | None | 0.010972225591703123 | 0.016515906883771553 | 0.014082038478294222 | 0.026278012976678557 |
| 5 | 0.046391361582157856 | 0.0067075424721699485 | 0.010672189505893723 | 0.00792155843585961 | 0.00897420589841433 | 0.013048248741068276 | None | 0.009888723390392798 | 0.01332242018387434 | 0.01628852751314251 | 0.01030865555291324 |
| 7 | 0.014179986801830653 | 0.002489376225232933 | 0.005262631159631594 | 0.005839254877480237 | 0.0032847516220848275 | 0.005448217446681274 | None | 0.0051187242338217274 | 0.003960779217929805 | 0.005262631159631603 | 0.005719084749787598 |TCRg
CD4
Relative expression
Relative expression
### Chart
| Category | | | | | | | | | | |
|---|---|---|---|---|---|---|---|---|---|---|
| 1 | 0.00176025480978678 | 0.0018997166941646217 | 0.0014598137193700576 | 0.0015864304616332728 | 0.0016310310926335343 | 1.1838585083926135e-07 | 0.001953125 | 0.0038792675603009177 | 0.0019667100587045312 | 0.0025771638882283094 |
| 5 | 0.001575472185980715 | 0.002152158429446504 | 0.0015537821948338258 | 0.0013715281989628864 | 0.0021822014415473026 | 1.5299628943954765e-07 | 0.002290693260218511 | 0.00359448301025342 | 0.0016537994382080606 | 0.002613139755441622 |
| 7 | 0.0005969923230918307 | 0.0014100871050024388 | 0.0007148855937234496 | 0.000502008704421908 | 0.0009303105449647826 | 9.549497098799033e-08 | 0.001076079214723252 | 0.0017360208616534574 | 0.001153313145927276 | 0.0013248089135231185 |
### Chart
| Category | | | | | | | | | | |
|---|---|---|---|---|---|---|---|---|---|---|
| 1 | 0.004186615088032399 | 0.0013620543616703184 | 0.0015112939390062388 | 0.0019396337801504623 | 0.0014699675267686226 | 0.001390674019137767 | 0.0021225290283712083 | 0.0022280541325555166 | 0.0027621358640099493 | 0.004518313218380027 |
| 5 | 0.0034720417233069156 | 0.001688549279898174 | 0.0032847516220848244 | 0.0012106520507216669 | 0.0011613350732448454 | 0.0013526459649520763 | 0.001700294068937741 | 0.0016768856180424871 | 0.0019396337801504588 | 0.001439716032510842 |
| 7 | 0.0009238844206304653 | 0.000719858016255421 | 0.0008384428090212449 | 0.0006442909720570778 | 0.0005969923230918307 | 0.0006487723701643119 | 0.0006857640994814444 | 0.0009111650307976642 | 0.0007715247186165798 | 0.0007987324790603327 |CD8
Perforin
Relative expression
Relative expression
