# Supplementary material for: Distribution of T Cells in Rainbow Trout (Oncorhynchus mykiss) Skin and Responsiveness to Viral Infection
Source: PLoS One. 2016 Jan 25;11(1):e0147477. doi: 10.1371/journal.pone.0147477 (PMC4726708; doi:10.1371/journal.pone.0147477)
Supplement: S2 Fig — Results obtained in Fig 2 for CD3, CD4, CD8, TCRα, TCRγ and perforin were plotted as correlative dispersion chart, for sections 1, 5 and 7. Correlated parameters were: CD3 vs TCRα, CD3 vs TCRγ, CD3 vs CD4, CD3 vs CD8, CD3 vs Perforin and CD8 vs Perforin. For each XY dispersion chart, a linear regression trend line is shown, together with the value of the correlation coefficient, denoted by R. Data are shown as the mean relative gene expression normalized to the transcription of the house-keeping gene EF-1α (n = 10). (PPTX) [file pone.0147477.s002.pptx]

## Slide 1
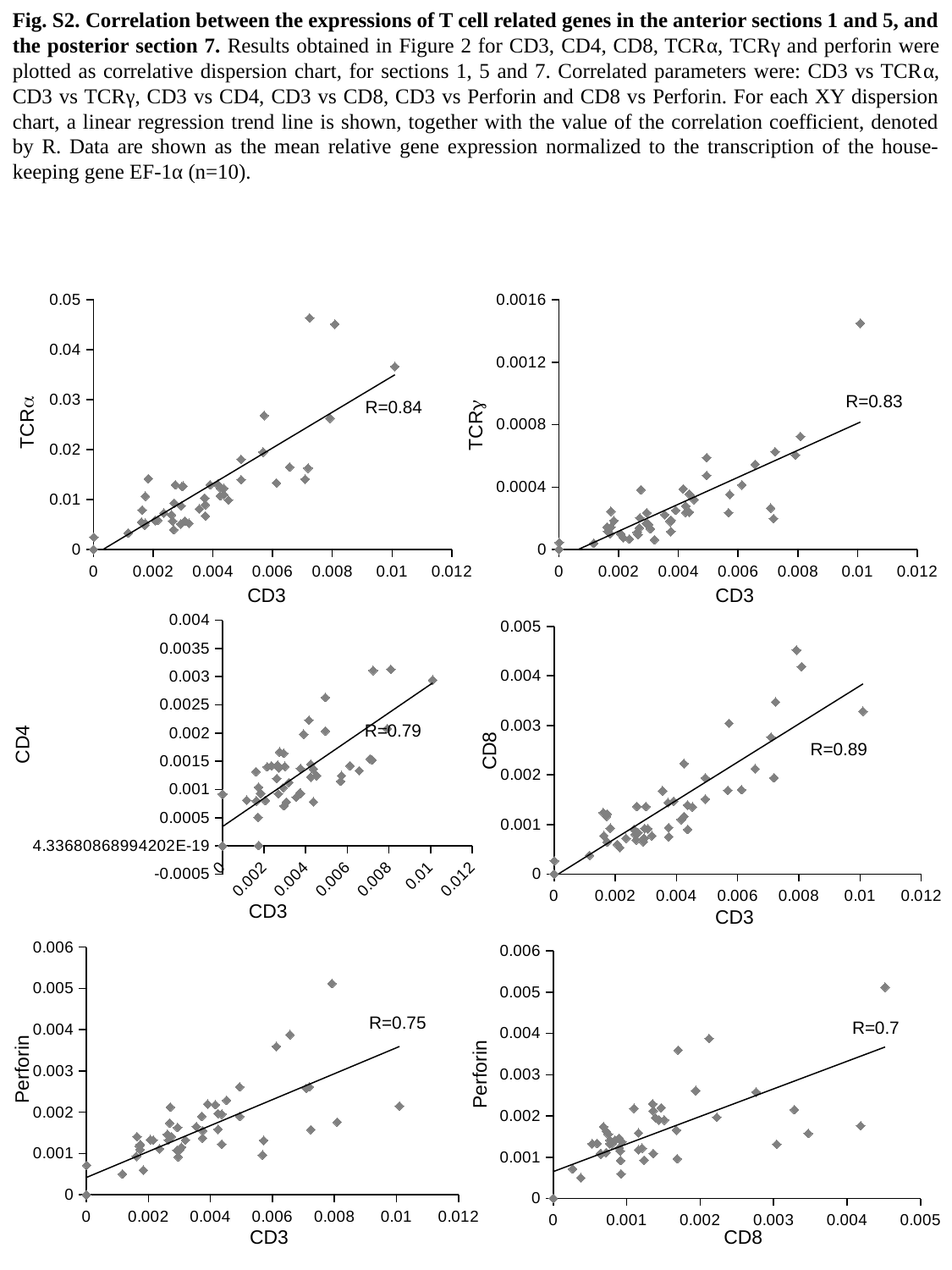

Fig. S2. Correlation between the expressions of T cell related genes in the anterior sections 1 and 5, and the posterior section 7. Results obtained in Figure 2 for CD3, CD4, CD8, TCRα, TCRγ and perforin were plotted as correlative dispersion chart, for sections 1, 5 and 7. Correlated parameters were: CD3 vs TCRα, CD3 vs TCRγ, CD3 vs CD4, CD3 vs CD8, CD3 vs Perforin and CD8 vs Perforin. For each XY dispersion chart, a linear regression trend line is shown, together with the value of the correlation coefficient, denoted by R. Data are shown as the mean relative gene expression normalized to the transcription of the house-keeping gene EF-1α (n=10).
### Chart
| Category | |
|---|---|
### Chart
| Category | |
|---|---|R=0.83
R=0.84
TCRa
TCRg
CD3
CD3
### Chart
| Category | |
|---|---|
### Chart
| Category | |
|---|---|R=0.79
CD4
R=0.89
CD8
CD3
CD3
### Chart
| Category | |
|---|---|
### Chart
| Category | |
|---|---|R=0.75
R=0.7
Perforin
Perforin
CD3
CD8
